# The relationship between mucosal immunity, nasopharyngeal carriage, asymptomatic transmission and the resurgence of *Bordetella pertussis*

**DOI:** 10.12688/f1000research.11654.1

**Published:** 2017-08-25

**Authors:** Christopher Gill, Pejman Rohani, Donald M Thea

**Affiliations:** 1 Centre for Global Health and Development , Boston University School of Public Health, Boston, Massachusetts, 02118, USA; 2 Department of Global Health, Boston University School of Public Health, Boston, Massachusetts, 02118, USA; 3 Department of Infectious Diseases College of Veterinary Medicine, Odum School of Ecology , University of Georgia, Athens, Georgia, 30602, USA

**Keywords:** whooping cough, acellular pertussis vaccine, bordetella pertussis

## Abstract

The incidence of whooping cough in the US has been rising slowly since the 1970s, but the pace of this has accelerated sharply since acellular pertussis vaccines replaced the earlier whole cell vaccines in the late 1990s. A similar trend occurred in many other countries, including the UK, Canada, Australia, Ireland, and Spain, following the switch to acellular vaccines. The key question is why. Two leading theories (short duration of protective immunologic persistence and evolutionary shifts in the pathogen to evade the vaccine) explain some but not all of these shifts, suggesting that other factors may also be important.

In this synthesis, we argue that sterilizing mucosal immunity that blocks or abbreviates the duration of nasopharyngeal carriage of *Bordetella pertussis* and impedes person-to-person transmission (including between asymptomatically infected individuals) is a critical factor in this dynamic. Moreover, we argue that the ability to induce such mucosal immunity is fundamentally what distinguishes whole cell and acellular pertussis vaccines and may be pivotal to understanding much of the resurgence of this disease in many countries that adopted acellular vaccines. Additionally, we offer the hypothesis that observed herd effects generated by acellular vaccines may reflect a modification of disease presentation leading to reduced potential for transmission by those already infected, as opposed to inducing resistance to infection among those who have been exposed.

## Introduction

*Bordetella pertussis*, the principal agent of whooping cough, infects millions of children around the world and kills many thousands, with severe and fatal cases concentrated among very young infants ^1– 3^. Pertussis is arguably the least well prevented of the common vaccine-preventable childhood diseases ^4^. Why this may be so reflects gaps in our understanding of the immunology and epidemiology of this pathogen, how these variables are affected by pertussis vaccines, and the degree to which they affect the transmissibility of *B. pertussis*.

**Figure 1.**
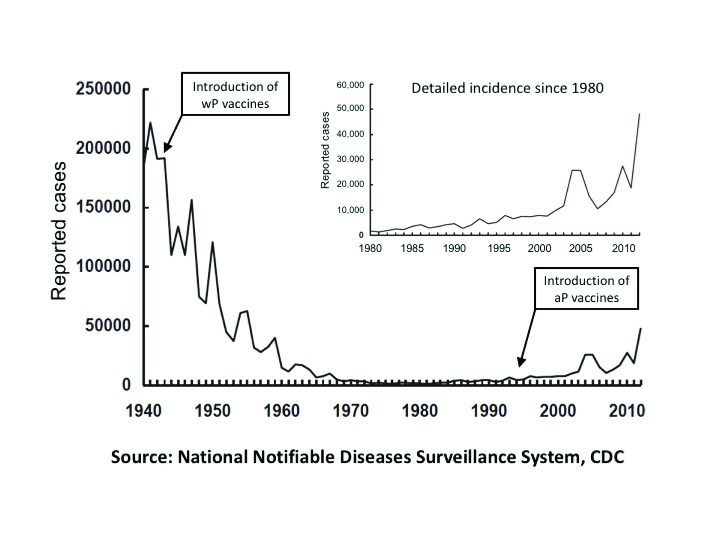
Trends in US pertussis incidence, 1940–2012. This figure depicts the annual per-capita incidence of pertussis infections in the US over the past seven decades, with the timing of the introduction of whole cell (wP) and acellular (aP) vaccines noted in annotations. The inset at the top right expands the details and readjusts the scale relevant to the period of rising incidence. As can be seen, pertussis rates were slowly rising since the 1970s but accelerated sharply following the transition to aP vaccines in 1996. The data are truncated as of 2010, but the rise of pertussis incidence has continued since that time.

A significant, but surprisingly under-examined, unknown is whether an asymptomatic infection state exists for *B. pertussis*, as is the case for many other bacterial respiratory pathogens, notably *Streptococcus pneumoniae*, *Haemophilus influenzae* type B (HiB), and *Neisseria meningitidis*. Each of these pathogens proved amenable to control using protein–polysaccharide conjugate vaccines. An essential attribute of these vaccines is their ability to induce robust mucosal immunity that interferes with or blocks these bacteria from colonizing the nasopharynx and propagating on the respiratory mucosa, irrespective of whether those events lead to clinical disease ^5– 7^. But does *B. pertussis* exist in an asymptomatic infection state also? Can pertussis transmit from asymptomatic individuals? Do pertussis vaccines interfere with these processes? And, if so, how? These are fundamental unresolved questions. The resurgence of pertussis in the US to its highest levels since the 1940s ^8, 9^ emphasizes the need for answers to these questions ( Figure 1).

**Figure 2.**
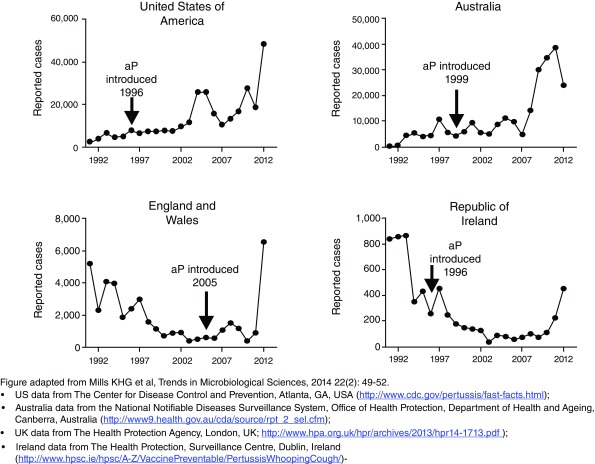
Changes in pertussis incidence relative to the introduction of acellular pertussis vaccines in the US, UK, Australia, and Ireland. Depicted are incidence data over time from the US, Australia, the United Kingdom (including Northern Ireland), and the Republic of Ireland. In each case, the introduction of acellular (aP) pertussis vaccines preceded an abrupt increase in disease incidence rates following a delay of 5–10 years. The declining incidence seen for the UK and Republic of Ireland from the early 1990s is likely explained by a reduction in whole cell pertussis vaccine uptake that occurred in the 1980s following several highly publicized adverse events that were attributed to pertussis vaccines.

There is increasing consensus that earlier whole cell pertussis (wP) vaccines impeded infections (not just clinical disease), generating herd immunity ^10, 11^, which, if we can extrapolate from the examples of Hib, pneumococcus, and meningococcus, could plausibly occur by interfering with *B. pertussis* colonization of the respiratory mucosa. It was subsequently assumed that subunit acellular (aP) vaccines would share this characteristic, particularly for aP vaccines that included the adhesion protein antigens pertactin (PRN), filamentous hemagglutinin (FHA), and fimbriae proteins 2 and 3 (Fim2/3). However, longitudinal nasopharyngeal (NP) sampling studies were not conducted to directly assess whether and to what degree either wP or aP vaccines interfered with *B. pertussis* carriage. That leaves us challenged to explain the surprising failure of several contemporary pertussis control strategies. Notably, why is whooping cough increasing in the US despite record rates of pertussis vaccine uptake, a pattern repeated in many other countries that switched from wP to aP vaccines ( Figure 2) ^12– 14^, and why has the strategy of vaccinating the household contacts of newborns (“cocooning”) failed to prevent pertussis among young infants ^15, 16^?

In this analysis, we examine the evidence surrounding possible explanations for the resurgence of pertussis and explore why many prior hypotheses fail to adequately explain current disease trends. From this, we offer a synthesis of old and new evidence that we believe points to a fundamentally different explanation in which the failure of aP vaccines to induce robust mucosal immunity plays a pivotal role. In addition, we offer a novel hypothesis for how aP vaccines may achieve some degree of herd immunity that is delinked from effects on mucosal immunity.

## The evidence

### Mucosal immunity and its link to herd immunity against respiratory pathogens

For the purposes of this manuscript, we define “mucosal immunity” as any process that leads to a protective immune response on a mucosal surface, even if the origin of that response may not reside locally within the mucosa itself. This definition therefore includes secreted antibody (IgA or IgG) that may derive from non-mucosal B-lymphocytes elsewhere in the body but which serve to interdict mucosal pathogens remotely.

Protein–polysaccharide conjugate vaccines targeting the trio of encapsulated respiratory pathogens HiB, *Streptococcus pneumoniae*, and *Neisseria meningitidis* have provided important insights into the mechanisms of vaccine-derived herd immunity ^17– 23^. In each case, the vaccines yielded steep reductions in disease among vaccinated and unvaccinated individuals, i.e. indirect herd effects, and, for each, the critical mechanism yielding herd effects proved to be a reduction in NP colonization rates, in turn mediated by sterilizing mucosal immunity ^23– 27^. To note, modeling studies suggest that at least half of the overall benefits of these vaccines rest on indirect, rather than direct, protection ^28^. In other words, herd immunity is not an incidental bonus feature of these vaccines; it is why they are so effective.

Obviously, *B. pertussis* disease differs substantially from disease caused by HiB, pneumococcus, or meningococcus, whose pathogenesis is mediated by inflammation and/or the interaction between the pathogen and biological systems within the host (e.g. complement and coagulation cascades and inflammatory pathways). By contrast, pertussis deals death at a distance through the elaboration of multiple toxins whose contributions to the pathogenesis of classical pertussis disease are complex and remain only partially understood ^29– 31^. Nonetheless, it is reasonable to postulate that pertussis' ability to adhere to, infect, and persist on the human respiratory mucosa could be an important factor in the overall epidemiology of pertussis. This theory seems supported by recent experimental mouse model data showing that *B. pertussis* can form biofilms that allow it to adhere to abiotic surfaces as well as to murine nasal and tracheal mucosa ^32^. Several cross-sectional studies in humans have also presented data compatible with the hypothesis of asymptomatic infection. But, while suggestive, these are not definitive proof for the simple fact that pertussis, as with all infectious diseases, has an incubation period. Without longitudinal follow up, there is no way to know whether an asymptomatic infected individual sampled “today” was not destined to have become symptomatic in coming days ^33, 34^.

### Failure of traditional explanations to account for the resurgence of pertussis

Compared to the pre-vaccine era, wP vaccines exerted a profound (~99%) reduction in pertussis incidence ( Figure 1) ^35^. But the newer-generation aP vaccines have been disappointing given that pertussis incidence has increased in many of the countries (though not all) where they replaced wP vaccines, including the US, Canada, Australia, Spain, Ireland, and the United Kingdom ^36– 40^. The key question is why.

Several hypotheses have been raised, of which the first three can be discounted:

1. **Detection bias:** traditional culture-based methods for diagnosing *B. pertussis* are insensitive because of the fastidious nature of the organism and because exposure to antibiotics can yield false negative results ^41, 42^. Such limitations could be overcome by using serologic markers of pertussis infection (such as a rise in anti-pertussis toxin [PTx] antibodies) or through the use of PCR, which may remain positive for weeks after the start of effective antibiotic therapy ^43^. The argument that PCR could result in an artefactual increase in incidence is intuitively appealing and could also explain the increased detection of milder/atypical presentations of pertussis ^44, 45^. Serologic markers are more difficult to interpret, since rises in titer may result from boosting after natural exposure absent disease or exposure to cross-reacting antigens.

**Figure 3.**
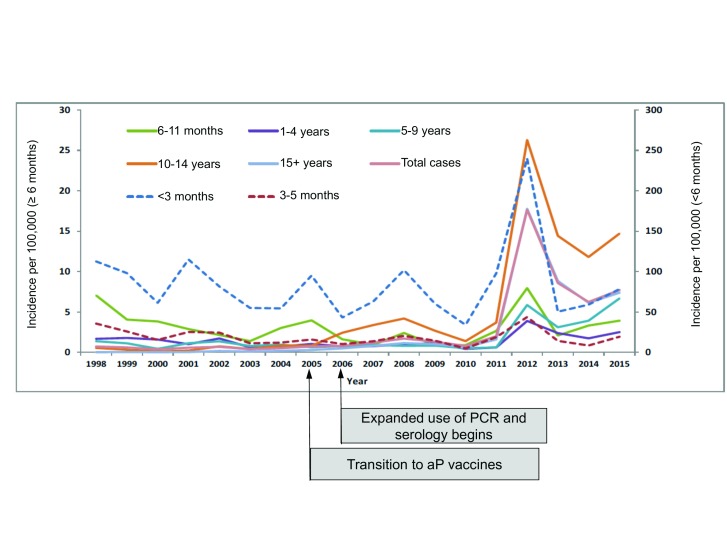
Pertussis incidence in the United Kingdom, by age group, 1998–2015. This figure presents data from the United Kingdom of pertussis incidence disaggregated by age categories over a 17-year period. The introduction of enhanced pertussis diagnostics and of the transition from whole cell to acellular (aP) pertussis vaccines are noted in annotations. To note, the scale representing incidence differs by age category (≥6 months versus <6 months of age). While there is year to year fluctuation in the incidence of pertussis, during the period 2011–2013, incidence rates spike. This abrupt increase cannot be easily explained as an artefact of enhanced diagnostics that were being phased in since 2006. A more parsimonious explanation is that this represents a nationwide epidemic of pertussis occurring after some delay from the transition to aP vaccines in 2005. PCR, polymerase chain reaction.

Regardless, improved ascertainment does not adequately explain the increases in severe pertussis and deaths reported among infants. It has been argued that severe or fatal disease in young infants should be relatively robust against detection bias, being inherently unlikely to evade diagnosis and reporting. Furthermore, the timing of the rising incidence is difficult to reconcile. For example, in the US, infant pertussis rates had been slowly increasing since the 1970s, preceding the switch to enhanced diagnostics ^11^. Conversely, the UK’s Health Protection Agency shifted to PCR and serologic diagnostics in 2006 ( Figure 3) ^46, 47^. In the years 2007–2009, the total number of cases reported increased modestly from ~100–300/year to 600–800/year, coincident and possibly explained by the phase in enhanced diagnostics. But that pattern shifted abruptly in 2011–2012 with over 1,000 and 9,000 cases in each year, respectively. The degree and rapidity of that increase is not readily explained by a phased adoption of new diagnostic techniques. Rather, it is more suggestive of a multi-year nationwide outbreak of pertussis that peaked in 2012. Enhanced diagnostics, and particularly PCR, may explain some increase in detection and very likely explains some of the shift towards the detection of milder cases, but the increase in pertussis incidence is real, not artefactual.

2. **Poor vaccine coverage**: aP vaccines were first introduced in the US in 1991 at limited scale for the fourth or fifth doses of an otherwise wP vaccine series, switching to the fully aP series in 1996. According to 2015 state by state statistics from the CDC, among US toddlers aged 19–35 months between 91 and 99% received three or more doses of DTaP and between 76 and 92% had received four or more doses ^48^; the introduction of the Tdap boosters to adolescents, older adults, and most recently pregnant women has further expanded coverage ^49^. The rise in pertussis in the US occurred despite historically high uptake of pertussis vaccines.

3. **Vaccine refusals:** as with measles, outbreaks of pertussis in unvaccinated or under-vaccinated populations have been well documented ^50^. Moreover, pertussis incidence increased among communities with high vaccine refusal rates, even among those who had been fully vaccinated, suggesting that the unvaccinated/under-vaccinated individuals pose a risk to the fully vaccinated members of those communities ^51^. Yet, given that US pertussis vaccine uptake is so high on average, the impact of vaccine-refusing communities must, by definition, be small and largely limited to those communities. It is difficult to see how this could significantly affect the overall increases seen across the general population in the US. Under-vaccination is an important factor in localized outbreaks but is insufficient to explain pertussis’ general and dispersed resurgence.

Evidence for two other hypotheses for the pertussis resurgence are more plausible:

4. **Poor persistence of anti-pertussis antibodies following aP vaccination**: many studies have documented rapid declines in pertussis antibodies, often to baseline concentrations, within as few as 2–3 years of the last aP vaccination (DTaP or Tdap), and these declines coincide with falling clinical efficacy ^52– 56^. Klein *et al*. estimated a 42% increase in the odds of acquiring clinical pertussis disease per year since the fifth dose of DTaP ^57^. Similarly, a meta-analysis by McGirr and Fisman estimated that the odds of pertussis disease increased by 33% for each year after the last aP vaccination, such that only 10% of fully vaccinated infants would remain protected after a median of 8.5 years ^58^.

Such data argue that poor immunologic persistence is an important factor in the resurgence of pertussis. However, it seems unlikely that all of this can be explained by that factor. Logically, with waning immunity, one would expect to see the rise proportional to increasing age groups, with the rise highest in adolescents, lower in school-aged children and toddlers, and lowest of all among vaccinated infants, which is what the UK data show ( Figure 3) ^46, 47^. The increases are bimodal, with the largest increases in incidence occurring among individuals aged 10 years and over and among infants aged <3 months. The rise among adolescents is well explained by poor immunological persistence after vaccination. By contrast, the increased incidence among infants too young to have been vaccinated is better explained by an overall rise in pertussis transmission, which may emanate from those older age groups. Immunologic persistence of protection elicited by aP vaccines has proven poorer than anticipated and very likely contributes to the resurgence of pertussis among older age groups. It is also likely that these older children act as a reservoir of infection that contributes to pertussis disease among very young infants.

5. **Leaky efficacy due to evolutionary shifts**: in contrast to wP vaccines, which expose the human immune system to a wide complement of *B. pertussis* antigens, aP vaccines include only a few or even one (i.e. pertussis toxoid-only vaccines) highly standardized antigen/s. While far less reactogenic, a potential tradeoff is that the vaccine is less adaptable to the plasticity of the *B. pertussis* genome.

Bart and colleagues recently published a series of phylogenetic analyses among global pertussis isolates collected spanning the pre-vaccine era, the wP vaccine era, and the aP vaccine era ^59, 60^. The introduction of aP vaccines, and to a lesser degree wP vaccines, appeared to coincide with shifts away from the specific allelic isoforms of the genes coding for most of the aP vaccine antigens. This included Fim2 and Fim3, PRN, and PTx. In each case, there was a shift to allelic isoforms genetically distinct from those contained in the aP vaccine (e.g. **Fim 3-1** to Fim 3-2 and 3-3; **PRN1** to PRN2; and **PTxA2** to PTxA1 [the aP vaccine alleles are shown in **bold font**]). In addition, the predominant promoter gene for PTx shifted from *PtxP1* to *PtxP3*, notable because PTxP3 hyper-expresses PTx relative to PTxP1, a further strategy for immune evasion simply by increasing the concentration of the antigenic target. Lastly, multiple studies have identified disease-causing pertussis isolates that ceased expressing one or more of the aP antigen genes entirely, a definitive strategy for evading antibodies targeting these proteins. This includes, singly or in combination, PRN, FHA, and, more recently, PTx ^61– 64^. Strains expressing PRN have virtually disappeared from the US ^65, 66^. While rare and dissociated from the profound lymphocytosis often seen in infant pertussis and mediated by PTx specifically ^61^, PTx-minus strains are surprising given prior assumptions that PTx was an obligatory virulence factor ^67^.

But, while provocative, the degree to which these shifts contributed to the pertussis resurgence is unclear. This uncertainty is justified by the continued effectiveness of aP vaccines in a number of countries where non-vaccine alleles have become quite common. For example, Sweden’s pertussis toxoid-only vaccine has retained efficacy for over 20 years ^68, 69^. It is also true that pertussis rates were slowly trending up in the US since the 1970s preceding the switch from wP to aP vaccines, though this has accelerated dramatically in the aP vaccine era ( Figure 1).

Another problem with this explanation is that the rise in population incidence observed in multiple countries seems to occur roughly 5–10 years after switching to aP vaccines and that the increases in incidence are often quite abrupt (see Figure 2). Presumably, the accumulation of non-vaccine antigen alleles among circulating strains of *B. pertussis* would be a gradual process, resulting in an incremental loss of efficacy with time. These abrupt step-wise transitions conflict with that expectation and do not account for why these seem to occur after a stereotypical delay from the wP to aP switch.

**Figure 4.**
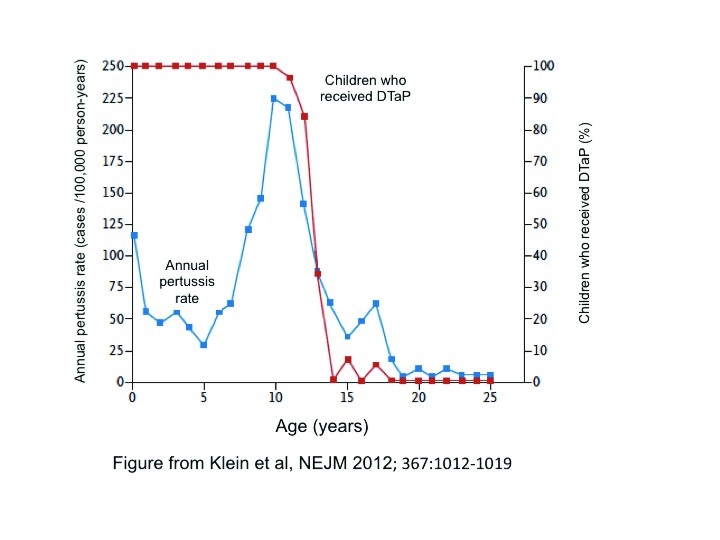
A natural experiment exploring the effect of infant exposure to acellular or whole cell pertussis vaccines and age-specific incidence of pertussis These data from the Kaiser Permanente health maintenance organization in Northern California reflect a natural experiment centered on the transition from whole cell to acellular pertussis vaccines in the US in the 1990s. These data were all collected over a narrow time window, and so the incidence by age should be understood in relation to what vaccine these individuals had received as infants. Those above the age of 15 could have received only whole cell pertussis vaccines, those under the age of 11 could have received only acellular pertussis vaccines, and those aged in between could have received a blended schedule of both vaccines, reflecting the transition period. The blue line depicts pertussis incidence as a function of age; the red line represents the proportion of each age stratum that received the acellular vaccine (100% of those vaccinated below the age of 11; ~0% of those aged 15 years and older). We make several observations: First, there is a decline in incidence from birth through the first year of life. This is likely explained by the induction of immunity through infant vaccination. Pertussis incidence then increases steadily through age 10. In light of what has been learned, this most likely reflects waning of acellular pertussis vaccine-induced immunity with time. Second, rather than continuing to rise with age, the incidence instead plummets above the age of 11 years, falling essentially to zero in those 15 years and older. It is remarkable to observe that receipt of any whole cell pertussis vaccines during infancy continues to exert such durable protection, to the extent that the 15 years and older birth cohort remain almost completely protected even decades out. This strongly argues that the immunologic effects resulting from whole cell and acellular pertussis vaccines are quite distinct. DTaP, diphtheria, tetanus, and acellular pertussis.

A good example of this leverages the natural experiment afforded by the US’ switch from wP to aP vaccines between 1991 and 1996. Klein *et al*. examined the incidence of PCR-confirmed pertussis illnesses as a function of age, which provided the natural experiment: anyone under the age of 11 years would mainly have received aP vaccines, those over the age of 15 could have received only wP vaccines, and those in between would have received a blended schedule of wP and aP vaccines. As shown in Figure 4, the incidence of pertussis increased with advancing age through 10 years, a trend well explained by waning immunity. But then incidence drops precipitously among the older cohorts who had received partly or fully wP vaccine schedules as infants, falling nearly to zero incidence among those 15 years and older. These data argue that wP vaccines were far more effective than aP vaccines (overall far lower incidence) and that they induced protective efficacy for significantly longer (i.e. beyond 15 years). Such striking differences also raise the following question: do the two classes of vaccine work immunologically in the same ways? We will return to this issue subsequently. Evolutionary shifts are likely in response to aP vaccines (and to a lesser degree wP vaccines) and plausibly contribute to the pertussis resurgence by degrading the efficacy of certain aP vaccines. But the extent of contribution is uncertain, and the abrupt rises in incidence do not fit well with evolution as the driving factor.

### Novel insights into pertussis transmission from modeling studies

In summary, persistence and evolutionary shifts likely contribute to the pertussis resurgence but seem insufficient to explain current disease trends. Something is still missing.

**Figure 5.**
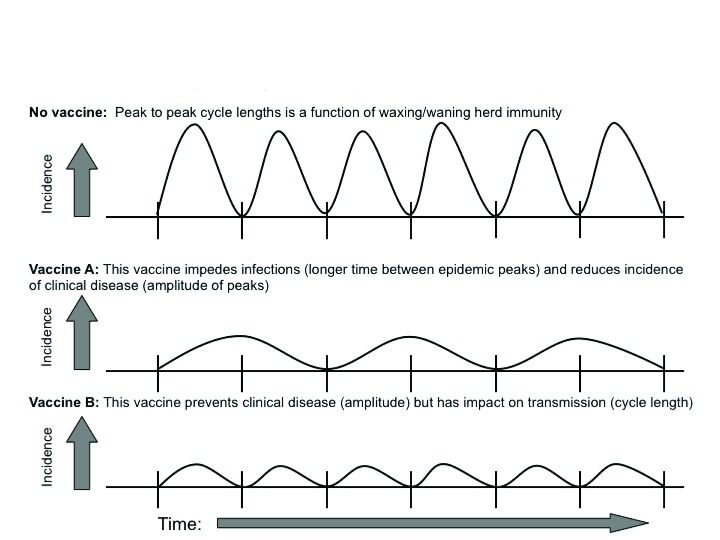
Effect of infection-blocking versus disease-preventing vaccines on the inter-epidemic cycle lengths of a hypothetical respiratory disease. These cartoons depict the effect on inter-epidemic cycle lengths by vaccines with different immunological effects in terms of whether they prevent infections (regardless of symptoms) as well as clinical disease (by definition symptomatic) or that only prevent clinical disease. At a population level, immunity waxes and wanes over time. With increased infection rates, the population acquires immunity and disease incidence subsequently falls. Over time, population immunity wanes (for example, because of the introduction of non-immune infants born into the population), leading to a resurgence of disease. The inter-epidemic cycle length is the average time between peaks in this cycle. The amplitude of each peak reflects the number of observed symptomatic cases in the population. •  Panel A depicts the base case absent any vaccination. For pertussis absent vaccination, the inter-epidemic cycle length has been estimated at between 3 and 5 years. •  Panel B depicts the effect of a vaccine that prevents infections and clinical disease. By blocking infections, the pace of spread through the population is slowed, leading to an extension of the inter-epidemic cycle length as well as a decline in amplitude of peaks due to prevention of clinical disease. •  Panel C depicts a vaccine that fails to block infection but effectively prevents clinical disease. This vaccine only reduces the amplitude of peaks but has no impact on period length, since transmission is not affected.

It has been argued that wP vaccines prevent disease but do not block infections. This conclusion rests on a single influential study published by Fine and Clarkson in the Lancet in 1982 ^70^. Their core argument concerned changes in the time period between pertussis epidemics and whether these expanded during the wP vaccine era (evidence that transmission was being impeded) (see examples in Figure 5). They found some lengthening of the inter-epidemic period but deemed this insufficient to suggest a decline in transmission and so concluded that wP vaccines did not prevent infections.

This conclusion is oft-cited and well engrained in the literature and official texts ^71, 72^ but is arguably flawed. Co-author Rohani and his team re-examined period lengths across multiple cities in England and Wales before and after wP vaccine introduction, with each city serving as its own control ^73^. When the data were aligned in this way, lengthening of inter-epidemic periods became more apparent, with average increases in each city of 1.5–2.5 years. This observation has since been replicated in 64 countries ^10, 74, 75^. In our view, the preponderance of evidence argues that wP vaccines impede disease transmission as well as prevent clinical disease ^76^.

This issue was readdressed by the modelers Althouse and Scarpino but now studying the question in reverse by focusing on the transition from wP to aP vaccines ^12^. Using a signal-processing technique called “wavelet analysis”, they found that the switch to aP vaccines preceded a contraction of the inter-epidemic period length, the opposite of introducing wP vaccines to a vaccine-naïve population.

The Althouse/Scarpino model further combined population-level incidence data and phylogenetic data mapping the genetic evolution of *B. pertussis* as a means to account for the burden of disease through observed contact linkages over space and time. Their surprising finding was a far higher pace of genetic mutation than could be accounted for via visible transmission pathways between known symptomatic cases. This excess genetic diversity implies longer chains of transmission between observed cases and therefore argues for the existence of asymptomatic transmission routes. This conclusion echoes that of the British epidemiologists Miller and Gay, writing in 2000, 4 years before the introduction of aP vaccines into the infant schedule ^77^. Disease among infants <3 months old (i.e. too young to have been vaccinated) increased sharply in the late 1970s, coincident with declines in wP coverage rates. As wP coverage improved in the 1980s, pertussis incidence fell, including among young infants. Surprisingly, though, the infant rates stabilized in the 100–200 cases/100,000 infants/year range, even as disease among older age groups fell almost to zero. Since those infants are unlikely to be their own reservoir for infection, a more plausible explanation was transmission from a sustained pool of asymptomatic older individuals in the population ^77^. This also emphasizes that even very high rates of wP vaccination may fail to completely interrupt pertussis transmission.

These models also offer a possible explanation for the surprising failure of “cocooning” to protect infants from pertussis. Cocooning refers to the strategy of administering Tdap to all household contacts (particularly the mother) of a newborn to prevent spread to the infant during the early months of life. Cocooning is logical but presupposes that aP vaccines prevent asymptomatic infections. Unfortunately, several controlled trials of cocooning in the US found no efficacy ^15, 16, 78^. These counterintuitive results conflict with expectations if aP vaccines block carriage and transmission but fit well if aP vaccines only prevent disease but have more limited ability to block infections.

### Recent experimental animal data show differential effects of aP and wP vaccines

Perhaps the most direct evidence of asymptomatic carriage and its implications emerged from an elegant series of experiments by Warfel and Merkel at the FDA using a newly developed infant baboon model. The key experiments in this series involved groups of infant baboons who were unvaccinated, vaccinated with aP vaccines, or vaccinated with wP vaccines and subsequently infected via exposure to aerosols of *B. pertussis* ^79^. In each case, vaccine-naïve baboons developed clinical illness, whereas aP- or wP-vaccinated animals remained asymptomatic (no coughing or lymphocytosis).

However, the animals also underwent serial NP sampling over the ensuing days and weeks, and here clear differences between the aP- and wP-vaccinated animals emerged. Among the wP-vaccinated baboons, NP carriage was detectable for a mean of 18 days (at low carriage densities) versus 30 and 35 days (at high densities) for naïve and aP-vaccinated animals, respectively. In fact, aP-vaccinated baboons eradicated NP colonization no more quickly than unvaccinated animals.

Follow-on experiments shed further light. Infant baboons immunized with aP vaccines and then infected with *B. pertussis* (but asymptomatic) were co-housed with vaccine-naïve baboons who had not been exposed to the *B. pertussis* aerosols: the unvaccinated cage mate animals also became infected, showing that aP-vaccinated animals remained contagious.

Approaching this in the opposite direction, the investigators infected a vaccine-naïve animal with pertussis and then co-housed it with both an aP-vaccinated animal and a second vaccine-naïve animal ^80^. Both of these animals acquired NP carriage at the same colonization density.

In a final experiment, female baboons were vaccinated with aP vaccines during pregnancy. Post-partum, the infant baboons were exposed to pertussis aerosols but were fully protected from clinical disease. However, all became colonized at similar densities as the infant baboons of unvaccinated mothers ^81^.

In summary, these animal experiments demonstrate that aP vaccines prevent disease but not infection and that this clinical protection is mediated by antibody, is transferable over the placenta, and can prevent disease post-partum in infants. But the aP vaccine-derived antibodies do not block colonization of the nasopharynx, allowing aP-vaccinated and passively protected animals to become infected and to propagate transmission. By contrast, wP-vaccinated animals diminished carriage in both bacterial burden and duration, clearing infections in half the time required by vaccine-naïve or aP-vaccinated animals. To further quantify this, they compared the cumulative area under the curve for *B. pertussis* shedding from the nasopharynx of vaccinated and infected animals: wP vaccines reduced the total burden of bacterial shedding by >1,000-fold compared with DTaP ^82^.

Empiric human evidence from a recent longitudinal study supports these interpretations. Looking at secondary attack rates among vaccine failures, the investigators calculated that wP vaccination reduced secondary infections by 86% compared with only 6% from aP vaccines ^83^.

### Recent immunologic insights and a possible missing link

As suggested by Klein’s natural experiment data ( Figure 4), the effects of wP and aP vaccines appear distinct. But what immunologic mechanism mediates these differences?

For decades, it was understood that naïve T-helper cells can differentiate into distinct lineages with distinct phenotypes and functional roles based on exposure to different combinations of antigenic stimuli, cytokine-mediated extracellular signaling from antigen presenting cells (APCs), and the specific combination of co-receptor molecules present at the interface between T-cells and the APC. The net result of these forces induces T-helper cells to adopt one of two functional phenotypes: TH1 or TH2 ^84^. TH1 cells are optimized for cell-mediated immunity targeting intracellular pathogens (such as tuberculosis), with actions mediated indirectly by T8 effector cells. By contrast, TH2 cells are optimized to stimulate B-cells for extracellular killing mediated primarily via antibodies and play a central role in allergic responses and controlling parasitic helminth infections. Yet, while helpful, this model has evident gaps, since it does not clearly explain a number of opportunistic conditions that characterize HIV/AIDS but lack a clear link to either TH1 or TH2 phenotypes.

More recently, a third TH cell developmental pathway was identified, whose inclusion seems to fill many of the gaps left by the TH1/TH2 paradigm. This was named TH17 in reference to the dominant cytokine (interleukin 17) released by APCs. TH17 lymphocytes direct neutrophil-mediated extracellular immune responses on mucosal surfaces, and deficits in TH17 manifest clinically as an inability to eradicate mucosal carriage and increased susceptibility to pathogens that colonize mucosal surfaces ^85^. Since HIV degrades TH17 and TH1 responses, whereas TH2 responses may actually increase in the setting of HIV/AIDS, this provides a far more satisfying explanation for such HIV-associated conditions as *Candida albicans* esophagitis, *Pneumocystis jirovecii* pneumonia, *Cryptococcus neoformans* meningitis, and invasive pneumococcal disease, conditions strongly associated with pathogen proliferation upon, and invasion across, mucosal surfaces. TH17 also proves directly relevant to pertussis ^86, 87^.

Warfel and Merkel assayed T-cell phenotypes in their infant baboons before and after infection with *B. pertussis*, or wP or aP vaccination. Pertussis infection triggered a purely TH17 response, whereas wP vaccination led to a predominantly TH17 response, with a lesser TH1 response. By contrast, aP vaccines induced only TH2 responses, which resulted in (mostly IgE) antibody generation and would not be expected to interfere with pathogens on mucosal surfaces ^88^.

These data suggest a definable immunologic mechanism distinguishing wP and aP vaccines that explains their differential effects on mucosal immunity and herd immunity and go a long way towards harmonizing the experimental and modeling data.

## Reconciling the exceptions

It must be acknowledged that the resurgence of pertussis seen in the US, the UK, and elsewhere has not occurred universally. Indeed, a recent analysis shows considerable heterogeneity in disease incidence among 20 countries that switched from wP to aP vaccinations ^76^. This emphasizes the complexity of the issue and the multiplicity of relevant factors that could influence incidence rates beyond a simple difference in vaccines: differential vaccine histories, pre-existing levels of herd immunity, birth rates, vaccine schedules, the specific make and composition of vaccines being employed, surveillance techniques, and the quality of data on which these estimates rest.

With that said, three countries merit particular mention because they are so often cited as counterfactual examples of the consequences of switching to aP vaccines. These are Japan (the first country to adopt aP vaccines globally), Italy, and Sweden.

The circumstances surrounding each of these examples is quite different from the US/UK/Canada/Spain/Australia contexts, where wP vaccines were phased out and aP vaccines phased in without interruption over a short period of transition (i.e. 1–2 years). To start with, Japan ceased using wP vaccines in 1974 following several deaths among wP-vaccinated infants. After a 7-year gap with no pertussis vaccines being used, Japan resumed using an aP vaccine in 1981 ^89^. Similarly, Sweden ceased all pertussis vaccinations in 1979, resuming with a monovalent aP vaccine after a 17-year hiatus in 1996 ^90^. In both cases, it is reasonable to assume that these gaps in coverage resulted in some loss of herd immunity achieved during the period of wP vaccine use, which makes it hard to draw meaningful comparisons about the shift from wP to aP vaccines. By contrast, in Italy, wP vaccines were in continuous use since the 1960s, but vaccine coverage was very poor (<40%). In response to rising incidence in the 1980s, aP vaccines were introduced in 1994 while simultaneously achieving very high rates (>90%) of vaccine coverage. But, while the pertussis burden fell sharply in the ‘90s, it is impossible to know whether incidence fell because of the change in vaccine or because of the increase in coverage ^91^.

## A hypothesis: a second pathway to herd immunity?

While the experimental and immunologic data suggest that aP vaccines fail to induce sterilizing mucosal immunity, the modeling data imply that aP vaccines probably do provide some degree of herd immunity. Co-author Rohani notes that mathematical models that include no impact on disease transmission cannot explain the observed epidemiologic data ^92^. For example, the monovalent PTx-only aP vaccine in Sweden led to impressive reductions in pertussis incidence outside of vaccinated populations, a signature of herd effect ^93, 94^.

But if the baboon data apply to humans and mucosal immunity is weak or absent following aP vaccines, how do we reconcile this apparent contradiction? Can we postulate another pathway to herd protection?

Several observations and some biologically plausible inferences may provide a clue. Warfel also showed that aerosol transmission occurred in the infant baboon model among animals housed in cages several meters away from an infected animal ^95^. This confirms that both short-range and long-range transmission of pertussis may occur. An undisputed benefit of aP vaccines is to prevent clinical disease, meaning that individuals do not suffer paroxysmal coughs. It is no great leap of faith to see paroxysmal coughing as a highly efficient way of generating infectious aerosols and enhancing long-distance transmission without requiring the kind of intimate close-contact between co-housed baboons – or that exists between a human mother and her baby. If so, aP vaccines would render infected individuals less contagious, even if they do not induce resistance to infection among those who were exposed. In this model, herd immunity reflects changes in the phenotype of disease expression reducing contagiousness (mediated by TH2 responses generating antibodies against PTx and other factors) rather than shifts in mucosal immunity (via TH17 responses) conferring resistance to exposure. This is a testable hypothesis.

## A proposed synthesis

We summarize the following key points in our discussion: 1) wP vaccinations curtailed pertussis disease and transmission; 2) pertussis rates increased with the introduction of aP vaccines in many (but not all) countries where there was a direct transition from wP to aP vaccinations; 3) modeling suggests that herd immunity is weaker for aP than wP vaccines; 4) the current burden of disease is not well explained by the disease that is observed but implies asymptomatic chains of transmission; 5) aP vaccination, or passively acquired antibodies resulting from aP vaccination, prevents symptomatic disease in animals but does not block infections; 6) transmission readily occurs between asymptomatic aP-vaccinated but infected animals to uninfected animals in close physical proximity; 7) wP and aP vaccines induce very different immunological responses in animals; 8) there are definable T-cell-mediated pathways (TH17) that confer mucosal immunity; 9) TH17 responses are robust following natural infection and wP vaccination but absent following aP vaccination, which only induces TH2 responses; and 10) the presence of TH17 responses aligns with a reduction in NP carriage in infant baboons.

In conclusion, the preponderance of available evidence now suggests that the list of plausible explanations for the resurgence of pertussis in the aP vaccination era goes beyond the “poor persistence” and “waning efficacy” of these vaccines to include an additional and likely pivotal factor: “lack of sterilizing mucosal immunity”. What is missing is direct evidence to validate this chain of logic, to go beyond the limitations of animal models, and to provide human data to support the hypotheses raised by the epidemiologists, experimentalists, and modelers. This issue would be greatly informed by carriage studies of *B. pertussis* among the general population, particularly around transitions in vaccine policy (such as future wP to aP transitions), and as an endpoint in clinical trials of any new third-generation pertussis vaccines.

## Where do we go now?

Fundamental aspects of pertussis epidemiology and immunology were left unexplained following the introduction of wP vaccines in the 1950s. The wP vaccines worked: disease rates plummeted, mortality fell, and the pertussis problem appeared largely solved. The fact that we did not know then, and still do not know now, how wP vaccines did this was inconvenient and has remained problematic in the aP vaccine era, since it created no clear immunologic surrogate by which to bridge these vaccine classes. Had carriage studies been conducted in parallel with wP introductions, then this might have provided supportive evidence that infections were being blocked. But carriage studies were not done. Rather, it was assumed that because wP vaccines appeared to confer herd immunity, they therefore blocked carriage. In hindsight and in light of subsequent evidence, that assumption was probably correct. It was subsequently assumed that aP vaccines, most of which include combinations of adhesion protein antigens (PRN, FHA, Fim2/3) that enable *B. pertussis* to bind to respiratory epithelium, would also block carriage. But, while logical, that assumption appears to have been incorrect.

In our view, we are approaching a critical decision point. In the US, pertussis rates and infant pertussis deaths are now at a 70-year high, and despite record uptake of aP vaccines, pertussis rates in the US have continued to rise, casting doubt on whether further increasing aP vaccine coverage can ever compensate for their fundamental limitations. Stanley Plotkin, among others, has offered several possible approaches to deal with this situation: (i) a return to the reactogenic wP vaccines (which would be challenging given our increasingly vaccine-averse population); (ii) integration of wP and aP vaccines in the infant schedules (also a difficult “sell”, notwithstanding that there are no longer any wP vaccines licensed in the US); (iii) attempts to broaden and enhance the immunogenicity of aP vaccines by adding in lipopolysaccharide or novel adjuvants; or (iv) developing new pertussis vaccines, including live-attenuated nasal vaccines that could be delivered directly at the NP mucosa specifically to enhance mucosal immunity ^96– 99^.

It is too early to predict how this will play out, but it seems essential that any future pertussis vaccine demonstrates its ability not just to prevent clinical disease but also to prevent infections, and showing this to be so will require carriage studies. The resurgence of pertussis in the aP vaccine era is evolving into a slow-moving global public health crisis. But, with the public’s trust in vaccines waning, this has also become a public relations crisis ^100^.

## References

[1] Robbins JB Schneerson R Trollfors B: Pertussis in developed countries. *Lancet.* 2002;360(9334):657–8. 10.1016/S0140-6736(02)09882-3 12241870

[2] De Serres G Duval B: Pertussis vaccination beyond childhood. *Lancet.* 2005;365(9464):1015–6. 10.1016/S0140-6736(05)71121-1 15781085

[3] Cutts FT Fine PE: Caution--mortality ratios ahead. *Lancet.* 2003;361(9376):2169–70. 10.1016/S0140-6736(03)13783-X 12842364

[4] Edwards KM: Overview of pertussis: focus on epidemiology, sources of infection, and long term protection after infant vaccination. *Pediatr Infect Dis J.* 2005;24(6 Suppl):S104–8. 10.1097/01.inf.0000166154.47013.47 15931137

[5] Roca A Hill PC Townend J: Effects of community-wide vaccination with PCV-7 on pneumococcal nasopharyngeal carriage in the Gambia: a cluster-randomized trial. *PLoS Med.* 2011;8(10):e1001107. 10.1371/journal.pmed.1001107 22028630 PMC3196470

[6] Lipsitch M: Vaccination against colonizing bacteria with multiple serotypes. *Proc Natl Acad Sci U S A.* 1997;94(12):6571–6. 10.1073/pnas.94.12.6571 9177259 PMC21091

[7] Maiden MC Stuart JM UK Meningococcal Carraige Group: Carriage of serogroup C meningococci 1 year after meningococcal C conjugate polysaccharide vaccination. *Lancet.* 2002;359(9320):1829–31. 10.1016/S0140-6736(02)08679-8 12044380

[8] Winter K Glaser C Watt J: Pertussis epidemic--California, 2014. *MMWR Morb Mortal Wkly Rep.* 2014;63(48):1129–32. 25474033 PMC4584602

[9] Centers for Disease Control and Prevention (CDC): Pertussis--United States, 2001–2003. *MMWR Morb Mortal Wkly Rep.* 2005;54(50):1283–6. 16371944

[10] Broutin H Viboud C Grenfell BT: Impact of vaccination and birth rate on the epidemiology of pertussis: a comparative study in 64 countries. *Proc Biol Sci.* 2010;277(1698):3239–45. 10.1098/rspb.2010.0994 20534609 PMC2981935

[11] Rohani P Drake JM: The decline and resurgence of pertussis in the US. *Epidemics.* 2011;3(3–4):183–8. 10.1016/j.epidem.2011.10.001 22094341

[12] Althouse BM Scarpino SV: Asymptomatic transmission and the resurgence of *Bordetella pertussis*. *BMC Med.* 2015;13:146. 10.1186/s12916-015-0382-8 26103968 PMC4482312

[13] Stokley S Cohn A Dorell C: Adolescent vaccination-coverage levels in the United States: 2006–2009. *Pediatrics.* 2011;128(6):1078–86. 10.1542/peds.2011-1048 22084326

[14] Stokley S Cohn A Jain N: Compliance with recommendations and opportunities for vaccination at ages 11 to 12 years: evaluation of the 2009 national immunization survey-teen. *Arch Pediatr Adolesc Med.* 2011;165(9):813–8. 10.1001/archpediatrics.2011.138 21893647

[15] Healy CM Rench MA Wootton SH: Evaluation of the impact of a pertussis cocooning program on infant pertussis infection. *Pediatr Infect Dis J.* 2015;34(1):22–6. 10.1097/INF.0000000000000486 24992123

[16] Castagnini LA Healy CM Rench MA: Impact of maternal postpartum tetanus and diphtheria toxoids and acellular pertussis immunization on infant pertussis infection. *Clin Infect Dis.* 2012;54(1):78–84. 10.1093/cid/cir765 22075790

[17] Moulton LH Chung S Croll J: Estimation of the indirect effect of *Haemophilus influenzae* type b conjugate vaccine in an American Indian population. *Int J Epidemiol.* 2000;29(4):753–6. 10.1093/ije/29.4.753 10922355

[18] Obaro S Adegbola R: The pneumococcus: carriage, disease and conjugate vaccines. *J Med Microbiol.* 2002;51(2):98–104. 10.1099/0022-1317-51-2-98 11863272

[19] O'Brien KL Dagan R: The potential indirect effect of conjugate pneumococcal vaccines. *Vaccine.* 2003;21(17–18):1815–25. 10.1016/S0264-410X(02)00807-1 12706665

[20] Trotter CL Andrews NJ Kaczmarski EB: Effectiveness of meningococcal serogroup C conjugate vaccine 4 years after introduction. *Lancet.* 2004;364(9431):365–7. 10.1016/S0140-6736(04)16725-1 15276396

[21] Zhang Q Finn A: Mucosal immunology of vaccines against pathogenic nasopharyngeal bacteria. *J Clin Pathol.* 2004;57(10):1015–21. 10.1136/jcp.2004.016253 15452151 PMC1770445

[22] Trotter CL Gay NJ Edmunds WJ: Dynamic models of meningococcal carriage, disease, and the impact of serogroup C conjugate vaccination. *Am J Epidemiol.* 2005;162(1):89–100. 10.1093/aje/kwi160 15961591

[23] Centers for Disease Control and Prevention (CDC): Direct and indirect effects of routine vaccination of children with 7-valent pneumococcal conjugate vaccine on incidence of invasive pneumococcal disease--United States, 1998–2003. *MMWR Morb Mortal Wkly Rep.* 2005;54(36):893–7. 16163262

[24] O'Brien KL Millar EV Zell ER: Effect of pneumococcal conjugate vaccine on nasopharyngeal colonization among immunized and unimmunized children in a community-randomized trial. *J Infect Dis.* 2007;196(8):1211–20. 10.1086/521833 17955440

[25] Stephens DS: Uncloaking the meningococcus: dynamics of carriage and disease. *Lancet.* 1999;353(9157):941–2. 10.1016/S0140-6736(98)00279-7 10459897

[26] Zhang Q Pettitt E Burkinshaw R: Mucosal immune responses to meningococcal conjugate polysaccharide vaccines in infants. *Pediatr Infect Dis J.* 2002;21(3):209–13. 10.1097/00006454-200203000-00010 12005084

[27] Dagan R Givon-Lavi N Zamir O: Effect of a nonavalent conjugate vaccine on carriage of antibiotic-resistant *Streptococcus pneumoniae* in day-care centers. *Pediatr Infect Dis J.* 2003;22(6):532–40. 10.1097/01.inf.0000069761.11093.c3 12799510

[28] Stephens DS: Protecting the herd: the remarkable effectiveness of the bacterial meningitis polysaccharide-protein conjugate vaccines in altering transmission dynamics. *Trans Am Clin Climatol Assoc.* 2011;122:115–23. 21686214 PMC3116338

[29] Hewlett EL Burns DL Cotter PA: Pertussis pathogenesis--what we know and what we don't know. *J Infect Dis.* 2014;209(7):982–5. 10.1093/infdis/jit639 24626533 PMC3952676

[30] Mattoo S Cherry JD: Molecular pathogenesis, epidemiology, and clinical manifestations of respiratory infections due to *Bordetella pertussis* and other *Bordetella* subspecies. *Clin Microbiol Rev.* 2005;18(2):326–82. 10.1128/CMR.18.2.326-382.2005 15831828 PMC1082800

[31] Fedele G Bianco M Ausiello CM: The virulence factors of *Bordetella pertussis*: talented modulators of host immune response. *Arch Immunol Ther Exp (Warsz).* 2013;61(6):445–57. 10.1007/s00005-013-0242-1 23955529

[32] Serra DO Conover MS Arnal L: FHA-mediated cell-substrate and cell-cell adhesions are critical for *Bordetella pertussis* biofilm formation on abiotic surfaces and in the mouse nose and the trachea. *PLoS One.* 2011;6(12):e28811. 10.1371/journal.pone.0028811 22216115 PMC3245231

[33] de Melker HE Versteegh FG Schellekens JF: The incidence of *Bordetella pertussis* infections estimated in the population from a combination of serological surveys. *J Infect.* 2006;53(2):106–13. 10.1016/j.jinf.2005.10.020 16352342

[34] Zhang Q Yin Z Li Y: Prevalence of asymptomatic *Bordetella pertussis* and *Bordetella parapertussis* infections among school children in China as determined by pooled real-time PCR: a cross-sectional study. *Scand J Infect Dis.* 2014;46(4):280–7. 10.3109/00365548.2013.878034 24520981

[35] Farizo KM Cochi SL Zell ER: Epidemiological features of pertussis in the United States, 1980–1989. *Clin Infect Dis.* 1992;14(3):708–19. 10.1093/clinids/14.3.708 1562663

[36] Amirthalingam G Gupta S Campbell H: Pertussis immunisation and control in England and Wales, 1957 to 2012: a historical review. *Euro Surveill.* 2013;18(38): pii: 20587. 10.2807/1560-7917.ES2013.18.38.20587 24084340

[37] Murphy JF: Pertussis has re-emerged. *Ir Med J.* 2012;105(8):260. 23155908

[38] Sizaire V Garrido-Estepa M Masa-Calles J: Increase of pertussis incidence in 2010 to 2012 after 12 years of low circulation in Spain. *Euro Surveill.* 2014;19(32): pii: 20875. 10.2807/1560-7917.ES2014.19.32.20875 25139074

[39] Spokes PJ Gilmour RE: NSW annual vaccine-preventable disease report, 2010. *N S W Public Health Bull.* 2011;22(9–10):171–8. 10.1071/NB11028 22060055

[40] Quinn HE McIntyre PB: The impact of adolescent pertussis immunization, 2004–2009: lessons from Australia. *Bull World Health Organ.* 2011;89(9):666–74. 10.2471/BLT.11.086538 21897487 PMC3165977

[41] Qin X Galanakis E Martin ET: Multitarget PCR for diagnosis of pertussis and its clinical implications. *J Clin Microbiol.* 2007;45(2):506–11. 10.1128/JCM.02042-06 17151210 PMC1829034

[42] Tatti KM Tondella ML: Utilization of multiple real-time PCR assays for the diagnosis of *Bordetella* spp. in clinical specimens. *Methods Mol Biol.* 2013;943:135–47. 10.1007/978-1-60327-353-4_9 23104287

[43] Stone BL Daly J Srivastava R: Duration of *Bordetella pertussis* Polymerase Chain Reaction Positivity in Confirmed Pertussis Illness. *J Pediatric Infect Dis Soc.* 2014;3(4):347–9. 10.1093/jpids/piu004 26625456

[44] Pimentel AM Baptista PN Ximenes RA: Pertussis may be the cause of prolonged cough in adolescents and adults in the interepidemic period. *Braz J Infect Dis.* 2015;19(1):43–6. 10.1016/j.bjid.2014.09.001 25452019 PMC9427331

[45] Cagney M McIntyre PB Heron L: The relationship between pertussis symptomatology, incidence and serology in adolescents. *Vaccine.* 2008;26(44):5547–53. 10.1016/j.vaccine.2008.08.009 18723066

[46] Public Health England: Incidence of laboratory-confirmed pertussis cases by age group, England 1998–2015.2016.

[47] Public Health England: Whooping cough (pertussis) statistics. Reference Source

[48] Centers for Disease Control and Prevention: Childhood Diphtheria toxoid, Tetanus toxoid, acellular Pertussis (DTaP) Vaccination Coverage 2015. Accessed 13 March,2017 Reference Source

[49] Centers for Disease Control and Prevention (CDC): Adult vaccination coverage--United States, 2010. *MMWR Morb Mortal Wkly Rep.* 2012;61(4):66–72. 22298302

[50] Glanz JM McClure DL Magid DJ: Parental refusal of pertussis vaccination is associated with an increased risk of pertussis infection in children. *Pediatrics.* 2009;123(6):1446–51. 10.1542/peds.2008-2150 19482753

[51] Phadke VK Bednarczyk RA Salmon DA: Association Between Vaccine Refusal and Vaccine-Preventable Diseases in the United States: A Review of Measles and Pertussis. *JAMA.* 2016;315(11):1149–58. 10.1001/jama.2016.1353 26978210 PMC5007135

[52] Barreto L Guasparini R Meekison W: Humoral immunity 5 years after booster immunization with an adolescent and adult formulation combined tetanus, diphtheria, and 5-component acellular pertussis vaccine. *Vaccine.* 2007;25(48):8172–9. 10.1016/j.vaccine.2007.09.031 17945400

[53] Witt MA Katz PH Witt DJ: Unexpectedly limited durability of immunity following acellular pertussis vaccination in preadolescents in a North American outbreak. *Clin Infect Dis.* 2012;54(12):1730–5. 10.1093/cid/cis287 22423127

[54] Weston W Messier M Friedland LR: Persistence of antibodies 3 years after booster vaccination of adults with combined acellular pertussis, diphtheria and tetanus toxoids vaccine. *Vaccine.* 2011;29(47):8483–6. 10.1016/j.vaccine.2011.09.063 21945698

[55] Giuliano M Mastrantonio P Giammanco A: Antibody responses and persistence in the two years after immunization with two acellular vaccines and one whole-cell vaccine against pertussis. *J Pediatr.* 1998;132(6):983–8. 10.1016/S0022-3476(98)70395-6 9627590

[56] Giuliano M Mastrantonio P Giammanco A: Antibody kinetics and long-term sero-prevalence in the Italian clinical trial of acellular pertussis vaccines. *Dev Biol Stand.* 1997;89:275–8. 9272360

[57] Klein NP Bartlett J Rowhani-Rahbar A: Waning protection after fifth dose of acellular pertussis vaccine in children. *N Engl J Med.* 2012;367(11):1012–9. 10.1056/NEJMoa1200850 22970945

[58] McGirr A Fisman DN: Duration of pertussis immunity after DTaP immunization: a meta-analysis. *Pediatrics.* 2015;135(2):331–43. 10.1542/peds.2014-1729 25560446

[59] Bart MJ van Gent M van der Heide HG: Comparative genomics of prevaccination and modern *Bordetella pertussis* strains. *BMC Genomics.* 2010;11:627. 10.1186/1471-2164-11-627 21070624 PMC3018138

[60] Bart MJ Harris SR Advani A: Global population structure and evolution of *Bordetella pertussis* and their relationship with vaccination. *MBio.* 2014;5(2):e01074. 10.1128/mBio.01074-14 24757216 PMC3994516

[61] Bouchez V Brun D Cantinelli T: First report and detailed characterization of *B. pertussis* isolates not expressing Pertussis Toxin or Pertactin. *Vaccine.* 2009;27(43):6034–41. 10.1016/j.vaccine.2009.07.074 19666155

[62] van Gent M Heuvelman CJ van der Heide HG: Analysis of *Bordetella pertussis* clinical isolates circulating in European countries during the period 1998–2012. *Eur J Clin Microbiol Infect Dis.* 2015;34(4):821–30. 10.1007/s10096-014-2297-2 25527446 PMC4365279

[63] Zeddeman A van Gent M Heuvelman CJ: Investigations into the emergence of pertactin-deficient *Bordetella pertussis* isolates in six European countries, 1996 to 2012. *Euro Surveill.* 2014;19(33): pii: 20881. 10.2807/1560-7917.ES2014.19.33.20881 25166348

[64] Bouchez V Hegerle N Strati F: New Data on Vaccine Antigen Deficient *Bordetella pertussis* Isolates. *Vaccines (Basel).* 2015;3(3):751–70. 10.3390/vaccines3030751 26389958 PMC4586476

[65] Martin SW Pawloski L Williams M: Pertactin-negative *Bordetella pertussis* strains: evidence for a possible selective advantage. *Clin Infect Dis.* 2015;60(2):223–7. 10.1093/cid/ciu788 25301209

[66] Queenan AM Cassiday PK Evangelista A: Pertactin-negative variants of *Bordetella pertussis* in the United States. *N Engl J Med.* 2013;368(6):583–4. 10.1056/NEJMc1209369 23388024 PMC5115783

[67] Locht C Coutte L Mielcarek N: The ins and outs of pertussis toxin. *FEBS J.* 2011;278(23):4668–82. 10.1111/j.1742-4658.2011.08237.x 21740523

[68] Advani A Gustafsson L Carlsson RM: Clinical outcome of pertussis in Sweden: association with pulsed-field gel electrophoresis profiles and serotype. *APMIS.* 2007;115(6):736–42. 10.1111/j.1600-0463.2007.apm_628.x 17550382

[69] Hallander HO Advani A Donnelly D: Shifts of *Bordetella pertussis* variants in Sweden from 1970 to 2003, during three periods marked by different vaccination programs. *J Clin Microbiol.* 2005;43(6):2856–65. 10.1128/JCM.43.6.2856-2865.2005 15956409 PMC1151881

[70] Fine PE Clarkson JA: The recurrence of whooping cough: possible implications for assessment of vaccine efficacy. *Lancet.* 1982;1(8273):666–9. 10.1016/S0140-6736(82)92214-0 6121976

[71] Edwards KM Decker MD: Pertussis vaccines. In: Plotkin S, ed. *Vaccines* Philadelphia PA: Elsevier;2013;447–492. 10.1016/B978-1-4557-0090-5.00030-6

[72] Cherry JD Heininger U: Pertussis and other *Bordetella* infections. *Feigin and Cherry’s Textbook of Pediatric Infectious Diseases* Philadelphia, PA: Saunders/Elsevier;2014; **129**:1616–1639.e12. Reference Source

[73] Rohani P Earn DJ Grenfell BT: Impact of immunisation on pertussis transmission in England and Wales. *Lancet.* 2000;355(9200):285–6. 10.1016/S0140-6736(99)04482-7 10675078

[74] Broutin H Mantilla-Beniers NB Simondon F: Epidemiological impact of vaccination on the dynamics of two childhood diseases in rural Senegal. *Microbes Infect.* 2005;7(4):593–9. 10.1016/j.micinf.2004.12.018 15820150

[75] Blackwood JC Cummings DA Broutin H: Deciphering the impacts of vaccination and immunity on pertussis epidemiology in Thailand. *Proc Natl Acad Sci U S A.* 2013;110(23):9595–600. 10.1073/pnas.1220908110 23690587 PMC3677483

[76] Domenech de Cellès M Magpantay FM King AA: The pertussis enigma: reconciling epidemiology, immunology and evolution. *Proc Biol Sci.* 2016;283(1822): pii: 20152309. 10.1098/rspb.2015.2309 26763701 PMC4721090

[77] Gay NJ Miller E: Pertussis transmission in England and Wales. *Lancet.* 2000;355(9214):1553–4. 10.1016/S0140-6736(05)74603-1 10801196

[78] Carcione D Regan AK Tracey L: The impact of parental postpartum pertussis vaccination on infection in infants: A population-based study of cocooning in Western Australia. *Vaccine.* 2015;33(42):5654–61. 10.1016/j.vaccine.2015.08.066 26320420

[79] Warfel JM Zimmerman LI Merkel TJ: Acellular pertussis vaccines protect against disease but fail to prevent infection and transmission in a nonhuman primate model. *Proc Natl Acad Sci U S A.* 2014;111(2):787–92. 10.1073/pnas.1314688110 24277828 PMC3896208

[80] Warfel JM Merkel TJ: The baboon model of pertussis: effective use and lessons for pertussis vaccines. *Expert Rev Vaccines.* 2014;13(10):1241–52. 10.1586/14760584.2014.946016 25182980

[81] Warfel JM Papin JF Wolf RF: Maternal and neonatal vaccination protects newborn baboons from pertussis infection. *J Infect Dis.* 2014;210(4):604–10. 10.1093/infdis/jiu090 24526741 PMC4133576

[82] Warfel JM Zimmerman LI Merkel TJ: Comparison of Three Whole-Cell Pertussis Vaccines in the Baboon Model of Pertussis. *Clin Vaccine Immunol.* 2015;23(1):47–54. 10.1128/CVI.00449-15 26561389 PMC4711092

[83] Préziosi MP Halloran ME: Effects of pertussis vaccination on transmission: vaccine efficacy for infectiousness. *Vaccine.* 2003;21(17–18):1853–61. 10.1016/S0264-410X(03)00007-0 12706669

[84] Mosmann TR Cherwinski H Bond MW: Two types of murine helper T cell clone. I. Definition according to profiles of lymphokine activities and secreted proteins. *J Immunol.* 1986;136(7):2348–57. 2419430

[85] Khader SA Gaffen SL Kolls JK: Th17 cells at the crossroads of innate and adaptive immunity against infectious diseases at the mucosa. *Mucosal Immunol.* 2009;2(5):403–11. 10.1038/mi.2009.100 19587639 PMC2811522

[86] Ross PJ Sutton CE Higgins S: Relative contribution of Th1 and Th17 cells in adaptive immunity to *Bordetella pertussis*: towards the rational design of an improved acellular pertussis vaccine. *PLoS Pathog.* 2013;9(4):e1003264. 10.1371/journal.ppat.1003264 23592988 PMC3617212

[87] Higgs R Higgins SC Ross PJ: Immunity to the respiratory pathogen *Bordetella pertussis*. *Mucosal Immunol.* 2012;5(5):485–500. 10.1038/mi.2012.54 22718262

[88] Warfel JM Merkel TJ: *Bordetella pertussis* infection induces a mucosal IL-17 response and long-lived Th17 and Th1 immune memory cells in nonhuman primates. *Mucosal Immunol.* 2013;6(4):787–96. 10.1038/mi.2012.117 23187316

[89] Watanabe M Nagai M: Acellular pertussis vaccines in Japan: past, present and future. *Expert Rev Vaccines.* 2005;4(2):173–84. 10.1586/14760584.4.2.173 15889991

[90] Carlsson RM von Segebaden K Bergstrom J: Surveillance of infant pertussis in Sweden 1998–2012; severity of disease in relation to the national vaccination programme. *Euro Surveill.* 2015;20(6): pii: 21032. 10.2807/1560-7917.ES2015.20.6.21032 25695476

[91] Gonfiantini MV Carloni E Gesualdo F: Epidemiology of pertussis in Italy: disease trends over the last century. *Euro Surveill.* 2014;19(40):20921. 10.2807/1560-7917.ES2014.19.40.20921 25323077

[92] Domenech de Cellès M Riolo MA Magpantay FM: Epidemiological evidence for herd immunity induced by acellular pertussis vaccines. *Proc Natl Acad Sci U S A.* 2014;111(7):E716–7. 10.1073/pnas.1323795111 24516173 PMC3932918

[93] Trollfors B Taranger J Lagergård T: Immunization of children with pertussis toxoid decreases spread of pertussis within the family. *Pediatr Infect Dis J.* 1998;17(3):196–9. 10.1097/00006454-199803000-00005 9535245

[94] Carlsson RM Trollfors B: Control of pertussis--lessons learnt from a 10-year surveillance programme in Sweden. *Vaccine.* 2009;27(42):5709–18. 10.1016/j.vaccine.2009.07.092 19679218

[95] Warfel JM Beren J Merkel TJ: Airborne transmission of *Bordetella pertussis*. *J Infect Dis.* 2012;206(6):902–6. 10.1093/infdis/jis443 22807521 PMC3501154

[96] Meade BD Plotkin SA Locht C: Possible options for new pertussis vaccines. *J Infect Dis.* 2014;209 Suppl 1:S24–7. 10.1093/infdis/jit531 24626868

[97] Plotkin SA: The pertussis problem. *Clin Infect Dis.* 2014;58(6):830–3. 10.1093/cid/cit934 24363332

[98] Mills KH Ross PJ Allen AC: Do we need a new vaccine to control the re-emergence of pertussis? *Trends Microbiol.* 2014;22(2):49–52. 10.1016/j.tim.2013.11.007 24485284

[99] Locht C: Live pertussis vaccines: will they protect against carriage and spread of pertussis? *Clin Microbiol Infect.* 2016;22 Suppl 5:S96–S102. 10.1016/j.cmi.2016.05.029 28341014

[100] Siddiqui M Salmon DA Omer SB: Epidemiology of vaccine hesitancy in the United States. *Hum Vaccin Immunother.* 2013;9(12):2643–8. 24247148 10.4161/hv.27243 PMC4162046

